# Sex differences in health-related quality of life among individuals at high risk of dementia

**DOI:** 10.1007/s41999-025-01278-w

**Published:** 2025-08-01

**Authors:** Ana Sofia Oliveira, Sílvia Lopes, Lara Noronha Ferreira, Vítor Tedim Cruz, Ana Rute Costa

**Affiliations:** 1 Local Health Unit Between Douro and Vouga, Santa Maria da Feira, Portugal; 2 NOVA National School of Public Health, NOVA University Lisbon, Lisbon, Portugal; 3 NOVA National School of Public Health, CHRC, REAL, CCAL, NOVA University Lisbon, Lisbon, Portugal; 4 Universidade do Algarve - ESGHT, Faro, Portugal; 5 Research Centre for Tourism, Sustainability and Well-Being (CinTurs), Faro, Portugal; 6 Centre for Health Studies and Research, Centre for Innovative Biomedicine and Biotechnology, University of Coimbra, Coimbra, Portugal; 7 EPIUnit ITR, Instituto de Saúde Pública da Universidade do Porto, Universidade do Porto, Porto, Portugal; 8 Serviço de Neurologia, Hospital Pedro Hispano, Unidade Local de Saúde de Matosinhos, Senhora da Hora, Portugal

**Keywords:** Quality of life, Cognitive decline, Dementia, Gender, Risk factors

## Abstract

**Purpose:**

Dementia represents an increasing challenge to health systems globally, with a notable impact on health-related quality of life (HRQoL). Nevertheless, the potential effect of sex on the relation between individuals’ characteristics and HRQoL, particularly in the early stages of this disease, remains unclear. Therefore, the present study aims to evaluate the association between sociodemographic, lifestyle and health-related factors with HRQoL among individuals at high risk of dementia, according to sex.

**Methods:**

This cross-sectional study was based on baseline data from the MIND-Matosinhos randomized controlled trial, targeting Portuguese adults at high risk of dementia [*n* = 207; 59.9% female; median age = 70.0 (interquartile range: 11)]. HRQoL was measured using the EQ-5D-5L. The associations between explanatory variables and HRQoL scores (dichotomized by the median) were quantified through odds ratios (OR) and 95% confidence intervals (CI), stratified by sex.

**Results:**

Overall, females reported lower HRQoL when compared with males [median (interquartile range): 0.875 (0.190) *vs.* 0.923 (0.129); *p* = 0.004]. Problems in mobility (43.6% *vs.* 27.7%; *p* = 0.021), pain/discomfort (71.8% *vs.* 44.6%; *p* < 0.001) and anxiety/depression (66.9% *vs.* 45.8%; *p* = 0.002), as well as the existence of any problem in five dimensions (7.3% *vs.* 3.6%; *p* = 0.004), were more frequently referred by females than males. A stronger association between poorer self-perceived health status and lower HRQoL was observed among females (OR = 8.75, 95% CI:3.64–21.03) compared to males (OR = 1.88, 95% CI:0.72–4.89; p for interaction = 0.020).

**Conclusion:**

Health status is associated with HRQoL, distinctively amongst males and females. These findings emphasize the need for sex-specific public health strategies to improve HRQoL in a vulnerable population of individuals at high risk of dementia.

**Supplementary Information:**

The online version contains supplementary material available at 10.1007/s41999-025-01278-w.

## Background

Worldwide, an overall growing burden of dementia has been observed during the last few decades [1]. Additionally, given the demographic shifts, an increasingly aging society, as well as a higher exposure to some risk factors, its incidence is expected to continue to grow [2]. This predicted increment in the burden of dementia brings substantial challenges to healthcare systems, particularly in countries facing considerably aged populations, such as Portugal. In fact, this country is becoming the fastest ageing country in Europe [3], and dementia prevalence is projected to double from 2020 to 2080 [4].

The quality of life (QoL) related to a specific disease is a crucial outcome, particularly for progressive conditions like cognitive decline and dementia, where the potential for long-term health improvements, such as reducing symptoms and prolonging survival, is limited [5, 6]. QoL is a subjective and multifaceted concept encompassing physical and mental well-being throughout an individual’s lifespan. More specifically, health-related quality of life (HRQoL) is a multidimensional measure of a person's subjective thoughts and feelings about the impact of their state of health on various aspects of their life, including the physical, social and psychological domains [7, 8].

There is strong evidence that HRQoL deteriorates over time as dementia progresses [9]. A systematic review of 19 studies focusing on older people with dementia concluded that functional impairment and depression are key factors influencing the HRQoL of these individuals [10], underlining the negative impact of physical and mental health. Nevertheless, it is still unclear how HRQoL is affected in the initial phases of cognitive decline and what are its main associated factors. Understanding these factors and their variation over time may contribute to planning adapted interventions to maintain or improve, when possible, the HRQoL of people living with cognitive impairment and dementia.

In addition, sex differences in both incidence and prevalence of dementia [11, 12] and levels of HRQoL are often assumed; however, contradictory results have been observed. Previous literature has reported consistently worse HRQoL among women [13, 14]; nevertheless, in several studies, demographic factors such as sex do not appear to be associated with the HRQoL of people with dementia [15, 16].

Therefore, this investigation aims to study the association between sociodemographic and health-related characteristics and HRQoL among individuals at high risk of dementia, according to sex.

## Methods

### Study design and participants

This cross-sectional study is based on baseline data from the “Multiple Interventions to Prevent Cognitive Decline “ project (MIND-Matosinhos), a randomized controlled study aiming to assess the effectiveness of non-pharmacological interventions to prevent cognitive decline, namely cognitive training, physical exercise, nutrition education, capacitation to deal with cognitive decline, and assessment and correction of hearing loss (Registration number: NCT05383443).

Participants met the following criteria: (1) age between 18 and 85 years; (2) score on the Montreal Cognitive Assessment (MoCA) equal to or higher than the validated cut-off points, defined as two standard deviations (SD) below the mean for the corresponding age and education in the Portuguese population [17]; (3) a high risk of developing dementia over the following 20 years based on the Cardiovascular Risk Factors, Aging and Dementia (CAIDE) [18] risk score (score ≥ 6 points); (4) at least four years of schooling. The exclusion criteria included medical contraindications for conducting the physical exercise, lack of autonomy in performing daily activities, previous diagnosis of dementia, and major physical or cognitive disability that hampers full participation in all planned interventions. For the present study, individuals evaluated in the baseline assessment were included (*n* = 207).

The MIND-Matosinhos project was approved by the Ethics Committee of the Local Health Unit of Matosinhos (Ref. 63/CES/JAS and 71/CES/JAS) and the Data Protection Officer of the Institute of Public Health of the University of Porto. The study was conducted according to the guidelines established by the Declaration of Helsinki, and all participants signed an informed consent form.

### Data collection

Participants’ sociodemographics, health-related characteristics, lifestyles, as well as HRQoL, were obtained in face-to-face interviews through structured questionnaires applied by trained interviewers. These interviews were performed in different places in Matosinhos city (e.g., social/municipal institutions offices, parish councils).

### Sociodemographic, health-related characteristics, and lifestyles

Sociodemographic data included sex, age, educational level, marital status, household income, and occupational status. Educational level was determined by the number of completed years of formal schooling and further defined as: 4 years, 5 to 9 years, or ≥ 10 years of schooling. Marital status was dichotomized into partnered (married or in a common-law marriage) and not partnered (single, widowed, divorced, or separated). Participants’ monthly household income was categorized into four categories, ranging from ≤ 1000 to > 2000€. Occupational status was recoded into two groups: employed (full-time, part-time employee, or freelancer) and non-employed (retired, unemployed, disability pensioner, pre-retirement, student, or domestic). The social support was measured through the Oslo Social Support Scale (OSSS-3), which is a self-reported tool containing three items that measure social support levels [19], namely: (1) number of close confidants; (2) perceived concern from others; and (3) the relationship with neighbours.

Previous diagnoses of selected diseases studied were hypertension, hypercholesterolemia, diabetes type II, cardiovascular diseases, cancer, respiratory diseases, musculoskeletal and connective tissue disorders, renal and urinary disorders, and gastrointestinal and hepatobiliary disorders. Those with ≥ 2 health conditions were considered with multimorbidity. Possible presence of cognitive impairment was defined as participants having a score on the MoCA between 1.5 and 2 standard deviations (SD) below the mean for the corresponding age and education in the Portuguese population. Self-perception of health status (very poor, poor, fair, good, and very good) was also collected. Additionally, anthropometric measurements were used to calculate the participants’ body mass index: normal (< 25.0 kg/m^2^), overweight (25.0–29.9 kg/m^2^) and obesity (≥ 30.0 kg/m^2^).

Regarding lifestyles, smoking habits (never/ever) were assessed. Alcohol intake was recorded as the frequency of consumption within the last year and was summarized as never or < 1x/month; 1x/month to <1x/day; ≥ 1x/day. Physical activity was measured using the Short Form of the International Physical Activity Questionnaire (IPAQ) for the Portuguese population [20], and data were converted into metabolic equivalent (MET) minutes/week, classifying participants as ‘high/moderate’ (≥ 600 MET min/week) or ‘low’ (< 600 MET min/week) [21]. The “Mediterranean Diet Adherence Screener” (MEDAS) [22] was used to assess adherence to the Mediterranean diet (≥ 10 points).

### HRQoL

HRQoL was assessed through the EQ-5D five-level (5L) version [23]. In this study, the Portuguese version of the EQ-5D-5L was used [24]. This self-administered instrument is recognized for its efficacy in quantifying an individual’s health status via a dual-assessment approach: a descriptive system and a visual analogue scale (EQ-VAS).

The EQ-5D-5L descriptive framework comprises five dimensions: mobility (MO), self-care (SC), usual activities (UA), pain/discomfort (PD), and anxiety/depression (AD). The MO dimension evaluates the participant's ambulatory capabilities; SC assesses the individual’s ability for personal hygiene and dressing; the UA dimension quantifies engagement in vocational, educational, domestic, familial, or recreational tasks; the PD dimension measures the intensity of physical discomfort; and the AD dimension evaluates psychological distress levels. Each dimension is rated on a scale of five levels: no problems, slight problems, moderate problems, severe problems and extreme problems. Patients indicate their health status by selecting, from among the five levels, the one that most accurately corresponds to each dimension. This selection generates a single-digit number representing the chosen level for that dimension. These digits from the five dimensions are combined to form a five-digit number, encapsulating the patient's overall health state. According to respondents’ EQ-5D-5L answers, a combined health utility score was calculated using the five-digit number, where 1 corresponded to full health and 0 to a health state equivalent to death. The responses to the five health dimensions were weighted using Portuguese general population preferences, combined for each dimension [24]. The weights represent differences in the five severity levels of each health dimension.

Furthermore, the EQ-VAS component of the instrument records an individual’s self-perceived health on a vertically oriented visual analogue scale, extending from 0 (denoting the poorest health conceivable) to 100 (indicating optimal health).

For the purposes of the present study, EQ-5D-5L and EQ-VAS total scores were dichotomized according to the median of the participants’ scores distribution; additionally, to evaluate the prevalence of having a least a slight problem in each dimension, answers were dichotomized in not having problems (no problems) and having problems (including slight, moderate, severe and extreme problems).

## Statistical analysis

Data were described using frequencies and percentages for categorical variables, and median and interquartile ranges (IQR) for continuous variables. All variables were compared by sex using the chi-square or Mann–Whitney tests, as applicable.

To estimate the association between sociodemographic, health-related characteristics and lifestyles with lower EQ-5D-5L and EQ-VAS scores, as well as having at least a slight problem on each of the five dimensions of the EQ-5D-5L, odds ratio (OR) and respective 95% confidence intervals (CI) were computed using logistic regression. The potential interaction of sex was assessed by including the corresponding interaction terms in the models, and the coefficients were combined to obtain strata-specific estimates. All models included age and years of schooling (continuous variables).

Statistical significance was set at p < 0.05. Analyses were performed using STATA® version 15.1 (College Station, TX: StataCorp LLC, 2017).

## Results

Table 1 shows the general characteristics of the participants. Of the 207 participants included in this study, 59.9% were females. Females were younger than males [median 69.5 (IQR = 10) *vs.* 73.0 (IQR = 11) years; *p* = 0.024), were less frequently partnered (58.5% *vs.* 95.2%; *p* < 0.001), and referred less often smoking habits (ever: 29.0% *vs.* 78.3%; *p* < 0.001) and daily alcoholic beverages consumption (25.6% *vs.* 68.9%; *p* < 0.001). Additionally, previous diagnosis of musculoskeletal and connective tissue disorders (55.4% *vs.* 18.0%; *p* < 0.001) and obesity (31.2% *vs.* 18.3%; *p* = 0.006) were more frequent among females when compared with males.

**Table 1:** General characteristics of the participants

	All participants	Females	Males	*p*-value
	*n* (%)^a^	*n* (%)^a^	*n* (%)^a^	
	207 (100.0)	124 (59.9)	83 (40.1)	
Sociodemographic characteristics				
Age (years), median (IQR)	70.0 (11)	69.5 (10)	73.0 (11)	**0.024**
Education (years)				
4	83 (40.1)	53 (42.7)	30 (36.1)	
5–9	68 (32.8)	34 (27.4)	34 (41.0)	
≥ 10	56 (27.0)	37 (29.8)	19 (22.9)	0.122
Marital status				
With partner	151 (73.3)	72 (58.5)	79 (95.2)	
Without partner	55 (26.7)	51 (41.5)	4 (4.8)	** < 0.001**
Occupational status				
Unemployed	182 (88.8)	108 (87.8)	74 (90.2)	
Employed	23 (11.2)	15 (12.2)	8 (9.8)	0.588
Household income (€/month)				
≤ 1000	53 (27.5)	37 (32.5)	16 (20.2)	
1001–1500	57 (29.5)	31 (27.2)	26 (32.9)	
1501–2000	38 (19.7)	20 (17.5)	18 (22.8)	
> 2000	45 (23.3)	26 (22.8)	19 (24.0)	0.292
Social support				
Poor	29 (14.6)	20 (17.0)	9 (11.2)	
Moderate	102 (51.5)	61 (51.7)	41 (51.2)	
Strong	67 (33.8)	37 (31.4)	30 (37.5)	0.451
Health-related characteristics				
Previous diagnosis of health conditions (yes)				
Hypertension	108 (52.2)	60 (48.4)	48 (57.8)	0.182
Hypercholesterolemia	130 (64.4)	82 (67.8)	48 (59.3)	0.216
Diabetes Type II	31 (15.3)	16 (13.2)	15 (18.3)	0.324
Cardiovascular diseases	49 (25.6)	24 (20.9)	25 (32.9)	0.063
Cancer	30 (14.6)	15 (12.1)	15 (18.3)	0.217
Respiratory diseases	59 (33.0)	38 (35.2)	21 (29.6)	0.435
Musculoskeletal and connective tissue disorders	75 (39.9)	61 (55.4)	14 (18.0)	** < 0.001**
Renal and urinary disorders	51 (27.9)	33 (30.3)	18 (24.3)	0.378
Gastrointestinal and hepatobiliary disorders	68 (37.6)	42 (37.8)	26 (37.1)	0.925
Multimorbidity	118 (79.2)	68 (80.0)	50 (78.3)	0.780
Possible presence of cognitive impairment	47 (22.7)	24 (19.4)	23 (27.7)	0.160
Self-perception of health status (fair to very poor)	134 (64.7)	82 (66.1)	52 (62.6)	0.608
Body mass index				
Normal	52 (25.5)	36 (29.5)	16 (19.5)	
Overweight	99 (48.5)	48 (39.3)	51 (62.2)	
Obesity	53 (26.0)	38 (31.2)	15 (18.3)	**0.006**
Lifestyles				
Adherence to the Mediterranean Diet	38 (118.8)	21 (17.1)	17 (21.5)	0.430
Smoking (ever)	101 (48.8)	36 (29.0)	65 (78.3)	** < 0.001**
Alcoholic beverages consumption (last 12 months)				
Never or < 1/month	49 (25.6)	42 (35.9)	7 (9.5)	
≥ 1/month to < 1/day	61 (31.9)	45 (38.5)	16 (21.6)	
≥ 1/day	81 (42.4)	30 (25.6)	51 (68.9)	** < 0.001**
Physical activity (low)	93 (45.2)	56 (45.5)	37 (44.6)	0.893

IQR, Interquartile Range

^a^ Except if otherwise specified

*Notes* The total may not add to the sample size of each group due to missing values and percentages may not total 100 due to rounding. Bold values represent statistically significant differences (*p* < 0.050)

**Fig. 1:**
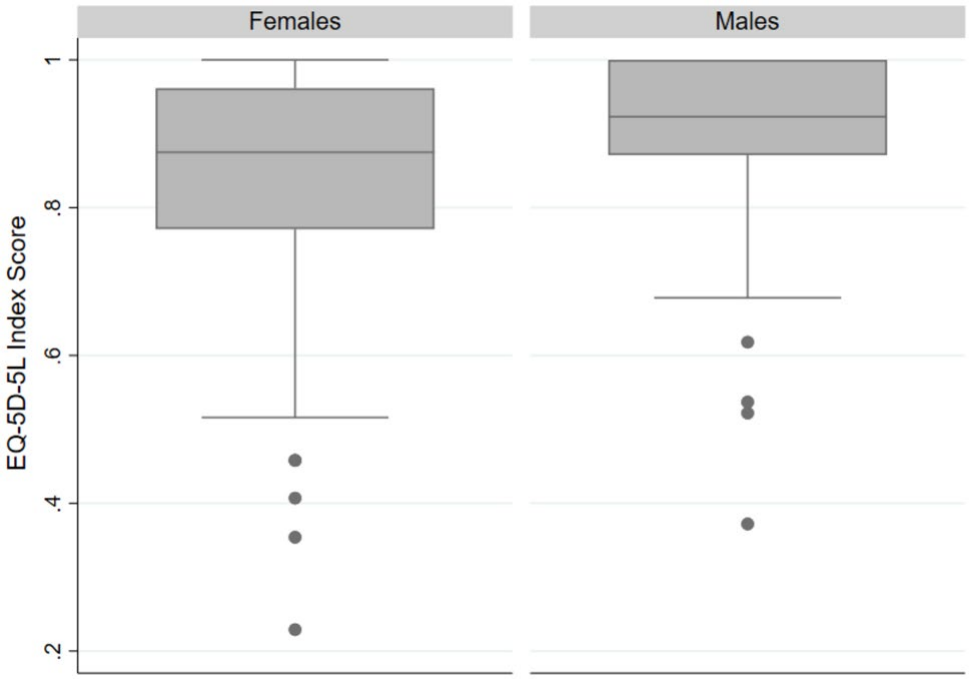
EQ-5D-5L index score, according to sex

**Fig. 2:**
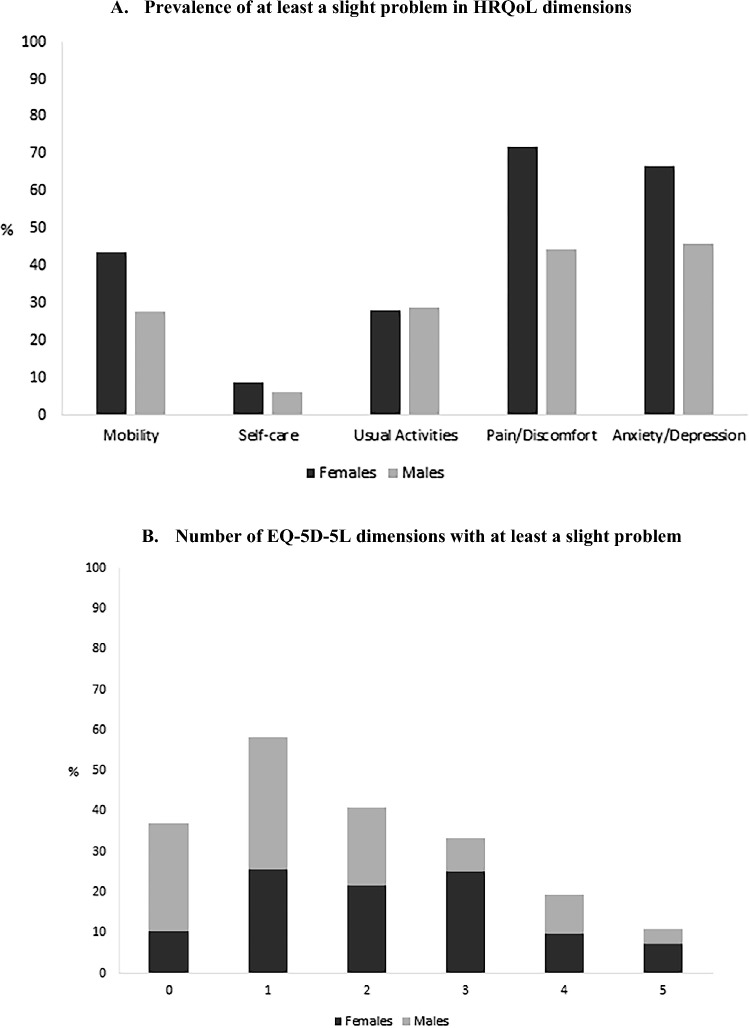
Prevalence of at least a slight problem in health-related quality of life (HRQoL) dimensions (**A**) and number of EQ-5D-5L dimensions with at least a slight problem (**B**), according to sex

Overall, females had a lower median HRQoL score than males [0.875 (IQR = 0.190) *vs.* 0.923 (IQR = 0.129); *p* = 0.004] (Fig. 1). However, no statistically significant differences among sexes were observed in the EQ-VAS median scores [females: 75 (IQR = 30) *vs.* males: 80 (IQR = 25); *p* = 0.340]. As observed in Fig. 2A, considering the five dimensions of the HRQoL, females were more likely to report at least a slight problem with MO (43.6% *vs.* 27.7%; *p* = 0.021), PD (71.8% *vs.* 44.6%; *p* < 0.001) and AD (66.9% *vs.* 45.8%; *p* = 0.002), when compared with males. Females also referred to the existence of problems in a higher number of dimensions (five dimensions with at least a slight problem: 7.3% *vs.* 3.6%; *p* = 0.004) (Fig. 2B).

Regarding the main characteristics associated with HRQoL levels, a stronger association between poorer self-perception of health status and lower HRQoL was observed among females (OR = 8.75, 95% CI: 3.64–21.03) compared to males (OR = 1.88, 95% CI: 0.72–4.89; *p* for interaction = 0.020) (Table 2). Furthermore, for both sexes, participants with obesity were more likely to present lower levels of HRQoL (females: OR = 7.91, 95% CI: 2.69–23.32; males: OR = 9.61, 95% CI: 1.56–58.97; *p* for interaction = 0.163). Despite the lack of statistically significant interaction of sex, females with previous diagnosis of cardiovascular diseases (OR = 3.06, 95% CI: 1.11–8.48), respiratory diseases (OR = 2.57, 95% CI: 1.11–5.95), renal and urinary disorders (OR = 4.11, 95% CI: 1.57–10.74) and multimorbidity (OR = 6.07, 95% CI: 1.56–23.55) presented more often lower levels of HRQoL whereas those with a higher household income (> €2000/month: OR = 0.26, 95% CI: 0.08–0.81) and daily consumption of alcoholic beverages (OR = 0.29, 95% CI: 0.11–0.79) were less likely to present this outcome (Table 2).

**Table 2:** Association between sociodemographics, health-related characteristics and lifestyles and lower levels of health-related quality of life (EQ-5D-5L score), according to sex

	Lower levels of HRQoL (EQ-5D-5L Score)		
	Females	Males	p for interaction
	OR (95% CI)^a^	OR (95% CI)^a^	
Sociodemographic characteristics			
Age (years)	0.99 (0.94–1.03)	1.06 (0.99–1.12)	0.086
Education (years)			
4	Reference	Reference	
5–9	0.52 (0.21–1.26)	0.82 (0.30–2.23)	
≥ 10	0.68 (0.29–1.63)	0.63 (0.19–2.16)	0.729
Marital status			
With partner	Reference	Reference	
Without partner	1.34 (0.64–2.79)	1.99 (0.26–15.51)	0.722
Occupational status			
Unemployed	Reference	Reference	
Employed	1.14 (0.33–3.93)	0.66 (0.11–4.06)	0.596
Household income (€/month)			
≤ 1000	Reference	Reference	
1001–1500	0.51 (0.19–1.39)	0.51 (0.14–1.82)	
1501–2000	0.39 (0.12–1.23)	0.45 (0.11–1.85)	
> 2000	**0.26 (0.08–0.81)**	0.51 (0.12–2.15)	0.837
Social support			
Poor	Reference	Reference	
Moderate	0.79 (0.28–2.22)	0.27 (0.06–1.26)	
Strong	1.28 (0.41–3.96)	0.30 (0.06–1.45)	0.338
Health-related characteristics			
Previous diagnosis of health conditions (Reference: no)			
Hypertension	1.31 (0.64–2.69)	1.61 (0.64–4.07)	0.730
Hypercholesterolemia	1.25 (0.58–2.70)	0.99 (0.39–2.51)	0.808
Diabetes Type II	2.47 (0.74–8.24)	3.07 (0.96–9.78)	0.798
Cardiovascular diseases	**3.06 (1.11–8.48)**	2.04 (0.76–5.48)	0.572
Cancer	2.36 (0.71–7.91)	1.51 (0.48–4.73)	0.599
Respiratory diseases	**2.57 (1.11–5.95)**	1.20 (0.41–3.48)	0.270
Musculoskeletal and connective tissue disorders	2.20 (0.99–4.87)	1.37 (0.42–4.49)	0.512
Renal and urinary disorders	**4.11 (1.57–10.74)**	2.92 (0.98–8.74)	0.648
Gastrointestinal and hepatobiliary disorders	1.33 (0.61–2.91)	1.94 (0.69–5.42)	0.567
Multimorbidity (Reference: no)	**6.08 (1.57–23.60)**	3.02 (0.59–15.35)	0.512
Possible presence of cognitive impairment (Reference: no)	1.08 (0.44–2.68)	1.04 (0.39–2.80)	0.956
Body mass index			
Normal	Reference	Reference	
Overweight	1.57 (0.64–3.87)	**6.34 (1.28–31.26)**	
Obesity	**7.91 (2.69–23.32)**	**9.61 (1.56–58.97)**	0.163
Self-perception of health status			
Good, very good	Reference	Reference	
Fair, poor, very poor	**8.75 (3.64–21.03)**	**1.88 (0.72–4.89)**	**0.020**
Lifestyles			
Adherence to the Mediterranean Diet (Reference: no)	0.80 (0.31–2.06)	0.28 (0.07–1.09)	0.219
Smoking			
Never	Reference	Reference	
Ever	1.44 (0.60–3.43)	1.25 (0.41–3.83)	0.849
Alcoholic beverages consumption (last 12 months)			
Never or < 1/month	Reference	Reference	
≥ 1/month to < 1/day	0.76 (0.32-1.84)	0.55 (0.03–9.40)	
≥ 1/day	**0.29 (0.11-0.79)**	0.83 (0.04–17.00)	0.450
Physical activity			
Moderate/high	Reference	Reference	
Low	1.66 (0.80–3.43)	2.09 (0.84–5.18)	0.696

CI, Confidence Interval; HRQoL, Health-related Quality of Life; OR, Odds Ratio

^a^ Adjusted for age and years of schooling (continuous variables)

*Note* Bold values represent *p*-values < 0.050

Concerning self-perceived health, EQ-VAS, both females and males with poorer self-perceived health status had lower scores of this outcome more often (females: OR = 7.76, 95% CI: 3.20–18.84; males: OR = 4.01, 95% CI: 1.50–10.70; p for interaction = 0.325) (Table 3). Additionally, females with cardiovascular diseases (OR = 4.01, 95% CI: 1.30–6.97), respiratory diseases (OR = 3.01, 95% CI: 1.30–6.97), and obesity (OR = 2.98, 95% CI: 1.15-7.74) were also more likely to refer lower levels of self-perceived health, while those taking alcoholic beverages every day presented this outcome less often (OR = 0.32, 95% CI: 0.12-0.86) (Table 3).

**Table 3:** Associations between sociodemographics, health-related characteristics and lifestyles and lower levels of self-perceived health (EQ-VAS), according to sex

	Lower levels of self-perceived health (EQ-VAS score)		
	Females	Males	p for interaction
	OR (95% CI)^a^	OR (95% CI)^a^	
Sociodemographic characteristics			
Age (years)	1.02 (0.97–1.07)	0.97 (0.92–1.03)	0.171
Education (years)			
4	Reference	Reference	
5–9	**0.32 (0.12–0.86)**	0.69 (0.26–1.86)	
≥ 10	0.63 (0.27–1.52)	0.86 (0.27–2.77)	0.256
Marital status			
With partner	Reference	Reference	
Without partner	1.49 (0.72–3.08)	4.12 (0.40–42.36)	0.415
Occupational status			
Unemployed	Reference	Reference	
Employed	0.79 (0.23–2.68)	0.64 (0.12–3.37)	0.835
Household income (€/month)			
≤ 1000	Reference	Reference	
1001–1500	0.57 (0.22–1.50)	0.85 (0.24–2.95)	
1501–2000	0.49 (0.16–1.50)	0.97 (0.25–3.78)	
> 2000	0.50 (0.17–1.50)	0.57 (0.14–2.35)	0.871
Social support			
Poor	Reference	Reference	
Moderate	0.79 (0.29–2.19)	0.23 (0.04–1.24)	
Strong	1.22 (0.41–3.67)	0.22 (0.04–1.25)	0.262
Health-related characteristics			
Previous diagnosis of health conditions (Reference: no)			
Hypertension	1.31 (0.64–2.66)	1.85 (0.75–4.55)	0.552
Hypercholesterolemia	0.95 (0.44–2.03)	1.39 (0.56–3.43)	0.525
Diabetes Type II	3.13 (0.94–10.45)	2.95 (0.90–9.66)	0.945
Cardiovascular diseases	**4.01 (4.45–11.07)**	2.39 (0.90–6.38)	0.472
Cancer	1.09 (0.37–3.21)	0.54 (0.17–1.78)	0.398
Respiratory diseases	**3.01 (1.30–6.97)**	0.80 (0.28–2.28)	0.052
Musculoskeletal and connective tissue disorders	1.85 (0.84–4.08)	1.03 (0.32–3.36)	0.415
Renal and urinary disorders	2.34 (0.98–5.56)	2.65 (0.89–7.90)	0.862
Gastrointestinal and hepatobiliary disorders	0.98 (0.45–2.11)	1.77 (0.66–4.75)	0.352
Multimorbidity (Reference: no)	1.90 (0.61–5.89)	3.15 (0.76-13.05)	0.580
Possible presence of cognitive impairment (Reference: no)	1.14 (0.46–2.79)	1.14 (0.43–2.99)	0.999
Body mass index			
Normal	Reference	Reference	
Overweight	1.13 (0.49–2.74)	1.45 (0.45–4.64)	
Obesity	**2.98 (1.15–7.74)**	1.99 (0.47–8.42)	0.685
Self-perception of health status			
Good, very good	Reference	Reference	
Fair, poor, very poor	**7.76 (3.20–18.84)**	**4.01 (1.50–10.70)**	0.325
Lifestyles			
Adherence to the Mediterranean Diet (Reference: no)	0.39 (0.14–1.04)	0.59 (0.19–1.79)	0.580
Smoking			
Never	Reference	Reference	
Ever	0.84 (0.36–1.97)	1.07 (0.37–3.10)	0.733
Alcoholic beverages consumption (last 12 months)			
Never or < 1/month	Reference	Reference	
≥ 1/month to < 1/day	0.85 (0.36–2.02)	0.79 (0.13–4.75)	
≥ 1/day	**0.32 (0.12–0.86)**	0.42 (0.83–2.13)	0.882
Physical activity			
Moderate/high	Reference	Reference	
Low	1.19 (0.59–2.43)	1.20 (0.50–2.87)	0.995

CI, Confidence Interval; OR, Odds Ratio

^a^ Adjusted for age and years of schooling (continuous variables)

*Note*: Bold values represent *p*-values < 0.050

The associations of different risk factors and each of the five dimensions of the EQ-5D-5L are presented in the supplementary material (Tables S1–S5). A statistically significant interaction of sex was observed regarding the association of poorer self-perceived health status and having PD problems (females: OR = 6.63, 95% CI: 2.77–15.86; males: OR = 1.13, 95% CI: 0.46–2.81; *p* for interaction = 0.006), as well as concerning the effect of low physical activity and having AD problems (females: OR = 0.62, 95%CI: 0.29–1.32; males: OR = 2.60, 95%CI: 1.05–6.39; *p* for interaction = 0.017) (Tables S4 and S5, respectively).

## Discussion

The present study has shown that, among individuals at high risk of dementia, females had lower overall median scores on the EQ-5D-5L and reported more problems related to MO, PD, and AD, when compared with males. The association between self-perceived health status and HRQoL differed according to sex, and, among females, previous diagnoses of specific diseases were positively related to lower levels of HRQoL.

The observed sex differences in HRQoL are consistent with the existing literature, which frequently reports that females generally experience lower HRQoL scores than males [25, 26]. This disparity can be attributed to several factors, including higher rates of chronic conditions [27] and greater psychosocial stress [28] among females. A longitudinal study on HRQoL in individuals with subjective cognitive decline and mild cognitive impairment also identified female sex as a predictor of lower HRQoL [29].

A detailed analysis of the EQ-5D-5L dimensions also revealed significant differences between the sexes. In our study, females were more likely to report problems in MO and AD dimensions, which is consistent with previous research [30, 31], where women also reported this type of problems more frequently compared to men, affecting both their physical and mental well-being. A possible explanation for this phenomenon is the association between musculoskeletal problems, which are more prevalent in women [32], leading to decreased MO [30]. In addition, previous studies indicate that women are disproportionately affected by these painful conditions and experience more persistent pain symptoms compared to men [32]. Concerning AD, the higher prevalence of depression in women can be attributed to biological and hormonal differences. Women are more susceptible to internalizing symptoms; additionally, hormonal fluctuations related to puberty, menstruation, pregnancy, and menopause may trigger depressive episodes. Hormonal transitions, particularly the decline in estrogen levels during the perimenopausal period, may increase this vulnerability. Additionally, depression has been recognised as a contributing factor to cognitive decline and dementia, and these hormonal changes may, therefore, play an indirect yet significant role in dementia risk. Experimental studies suggest that estrogen may exert protective effects on brain regions such as the hippocampus and on neurotransmitter systems involved in mood regulation. These mechanisms support the relevance of integrating sex-specific factors in strategies to prevent cognitive deterioration [33].

Although in the present study we observed similar scores on the EQ-VAS, the lower EQ-5D-5L index scores among females suggest that this group may have a more complex interaction of health problems affecting specific aspects of their HRQoL, which is not fully captured by the global self-assessment provided by the EQ-VAS. However, this difference may also be related to the fact that the EQ-VAS requires a quantification from 0 to 100, which can be more subjective to respond to when compared to the multiple-choice questions in the EQ-5D-5L dimensions. These findings underline the importance of multidimensional assessment tools such as the EQ-5D-5L to identify and address specific areas where interventions are needed.

Regarding specifically the association between sociodemographic factors and HRQoL, the influence of household income proved to be significant, suggesting economic status as a crucial factor for maintaining better HRQoL, a trend observed in multiple health studies [34, 35]; this generally provides better access to healthcare, directly influences living conditions, health literacy, and tends to reduce health risks due to greater awareness and resources to choose healthier lifestyles.

Cardiovascular diseases [36], respiratory diseases [37], renal and urinary disorders [38], and multimorbidity [39] were also associated with lower HRQoL in females, as observed in previous studies. This association is underscored by the rising prevalence of chronic diseases, which pose a significant public health challenge, particularly for women [40], and serve as major predictors of their HRQoL [41]. In both males and females, the significant association of obesity with lower HRQoL aligns with existing literature, which has consistently shown obesity to be a relevant predictor of lower HRQoL [42, 43], whether due to the physical complications it entails or the greater propensity to develop psychological problems resulting from social stigmatization and discrimination.

Furthermore, poor self-perceived health status was previously associated with lower HRQoL, particularly in females [44]. The stronger observed association between self-perceived health status and HRQoL in females may be due to differences in how the two sexes interpret and respond to the distinct categories of health status. Females may be more expressive about their health problems, which leads to lower HRQoL scores. In addition, social and cultural factors may influence the way females report their health status, possibly perceiving and communicating their health problems more acutely than males. Despite self-perceived health status and QoL addressing similar health-related issues, females tend to respond differently, as evidenced by the stronger association of self-perceived health status with HRQoL among women. The self-perceived health status question may capture aspects of their health concerns that are also reflected in their responses to HRQoL dimensions.

In our study, daily alcohol consumption was negatively associated with lower HRQoL. Although this finding may seem unexpected, it is consistent with previous research suggesting that light-to-moderate alcohol intake may have a protective effect on subjective well-being and cognitive function in older adults [45, 46]. Nonetheless, these results should be interpreted with caution due to the cross-sectional nature of our data, as well as the lack of detailed information regarding the type and quantity of alcohol consumed. Further longitudinal research is needed to explore these associations more comprehensively.

The present study provides a comprehensive assessment of HRQoL in a specific population at high risk of dementia using a well-validated instrument like the EQ-5D-5L. This tool allows for precise measurement across various health dimensions, enhancing the robustness of the findings. The study focuses on individuals with an increased vulnerability to dementia and their perception of health status, an area that remains under-researched. By addressing this critical gap, this work provides valuable insights into how various health and sociodemographic factors influence QoL in this specific and susceptible group. Additionally, the detailed stratification by sex and the examination of a wide range of variables offer a nuanced understanding of the factors influencing HRQoL.

Despite its strengths, this study has several limitations that should be highlighted. One limitation is the potential data bias due to the recruitment method. Participants of this study are primarily interested in taking part in a clinical trial regarding non-pharmacological strategies to prevent cognitive decline, which may hamper the generalization of these results. Additionally, a significant proportion of male participants attended the study primarily accompanied by their female partners, which may justify the higher proportion of married males compared to females. As a result, the HRQoL scores for males might not be entirely representative of the broader population at high risk for dementia. Furthermore, the low sample size, particularly of males, may have hindered the observation of other statistically significant results.

The cross-sectional design of this study limits the ability to draw causal inferences about the relationships between the variables studied. Therefore, longitudinal studies are needed to explore how HRQoL evolves over time in individuals at high risk of dementia. The reliance on self-reported data for HRQoL and health conditions could introduce bias, as participants might underreport or overreport their health status. This limitation could affect the accuracy of the associations found between health conditions and HRQoL. Additionally, the dichotomization of HRQoL scores was adopted to support statistical modelling, but we acknowledge this may reduce sensitivity to intermediate levels of impairment and mask important variability. Additionally, ceiling effects were observed in our sample, limiting the instrument’s ability to differentiate between individuals with relatively high HRQoL. Moreover, the study population was specific to a particular geographic region in Portugal, which might limit the generalizability of the findings to other populations with different sociodemographic and cultural characteristics. Future studies should aim to include more diverse populations to enhance the external validity of the results.

Considering the sex-specific disparities observed, public health interventions should include targeted support for females who are disproportionately affected by MO issues, chronic pain, and mental health conditions. Furthermore, longitudinal studies should be conducted to better understand the evolution of HRQoL over time in high-risk populations and to develop effective interventions that can be widely implemented. Besides enhancing the HRQoL, these strategies may contribute to reducing the prevalence of major risk factors for dementia.

## Conclusion

This study has shown the existence of sex differences in HRQoL among individuals at high risk of dementia. The findings indicate that females reported lower overall HRQoL scores than males, with more frequent problems in MO, PD, and AD dimensions. These disparities underscore the necessity for sex-specific public health strategies aimed at improving HRQoL in this vulnerable population. Tailored interventions that address the unique health challenges faced by females, such as chronic pain and mental health support, are essential for mitigating these differences. Additionally, the association between poorer self-perceived health status and lower HRQoL, particularly among females, suggests that enhancing self-perception of health through targeted education and support programs could be beneficial. These findings highlight the importance of implementing preventive strategies tailored to sex-specific differences. Although our sample included mainly older adults, the impact of such interventions may be more pronounced if initiated earlier in life. Furthermore, future research should focus on longitudinal studies to better understand the evolution of HRQoL over time in high-risk populations and to develop effective interventions that can be widely implemented.

## Supplementary Information

Below is the link to the electronic supplementary material.

Supplementary file1 (DOCX 42 kb)

## Data Availability

Due to the sensitive nature of the research, supporting data is not available.
