# Supplementary material for: Sex differences in health-related quality of life among individuals at high risk of dementia
Source: Eur Geriatr Med. 2025 Aug 1;16(6):2169–80. doi: 10.1007/s41999-025-01278-w (PMC12743678; doi:10.1007/s41999-025-01278-w)
Supplement: Supplementary file 1 — Supplementary file1 (DOCX 42 kb) [file 41999_2025_1278_MOESM1_ESM.docx]

**Supplementary material**

**Table S1** – Association between sociodemographics, health-related characteristics and lifestyles and having any problem in the mobility dimension, according to sex.

|  | **Any problem in the mobility dimension** | | |
| --- | --- | --- | --- |
|  | **Females** | **Males** | **p for**  **interaction** |
|  | OR (95% CI)^a^ | OR (95% CI)^a^ |  |
| **Sociodemographic characteristics** |  |  |  |
| Age (years) | 1.02 (0.97-1.08) | 1.05 (0.98-1.12) | 0.603 |
| Education (years) |  |  |  |
| 4 | Reference | Reference | 0.767 |
| 5-9 | 0.44 (0.18-1.10) | 0.47 (0.16-1.40) |  |
| ≥10 | 0.56 (0.23-1.34) | 0.32 (0.08-1.38) |  |
| Marital status |  |  |  |
| With partner | Reference | Reference | 0.237 |
| Without partner | 1.12 (0.54-2.36) | 4.41 (0.52-37.58) |  |
| Occupational status |  |  |  |
| Unemployed | Reference | Reference | 0.679 |
| Employed | 1.36 (0.36-5.11) | 2.11 (0.33-13.61) |  |
| Household income (€/month) |  |  |  |
| ≤1000 | Reference | Reference | 0.880 |
| 1001-1500 | 1.01 (0.38-2.68) | 0.71 (0.19-2.69) |  |
| 1501-2000 | 0.54 (0.17-1.77) | 0.30 (0.06-1.54) |  |
| >2000 | 0.54 (0.17-1.70) | 0.62 (0.13-2.86) |  |
| Social support |  |  |  |
| Poor | Reference | Reference | 0.458 |
| Moderate | 0.86 (0.31-2.41) | 0.29 (0.06-1.33) |  |
| Strong | 0.82 (0.27-2.50) | 0.26 (0.05-1.27) |  |
| **Health-related characteristics** |  |  |  |
| Previous diagnosis of health conditions (Reference: no) |  |  |  |
| Hypertension | 1.91 (0.92-3.97) | 2.340 (0.82-7.04) | 0.728 |
| Hypercholesterolemia | 1.48 (0.67-3.30) | 0.88 (0.32-2.42) | 0.426 |
| Diabetes Type II | 2.81 (0.90-8.82) | 3.08 (0.94-10.07) | 0.914 |
| Cardiovascular diseases | **2.82 (1.09-7.35)** | 1.89 (0.65-5.46) | 0.581 |
| Cancer | 2.25 (0.74-6.88) | 2.85 (0.87-9.28) | 0.778 |
| Respiratory diseases | **2.32 (1.01-5.32)** | 0.51 (0.14-1.82) | 0.051 |
| Musculoskeletal and connective tissue disorders | 1.91 (0.84-4.33) | 0.70 (0.17-2.85) | 0.223 |
| Renal and urinary disorders | **3.22 (1.35-7.71)** | 2.00 (0.64-6.26) | 0.515 |
| Gastrointestinal and hepatobiliary disorders | 1.06 (0.48-2.33) | 1.47 (0.49-4.44) | 0.634 |
| Multimorbidity (Reference: no) | **11.60 (1.44-93.71)** | 4.84 (0.57-41.01) | 0.565 |
| Possible presence of cognitive impairment (Reference: no) | 0.86 (0.34-2.17) | 0.89 (0.29-2.68) | 0.969 |
| Body mass index |  |  |  |
| Normal | Reference | Reference |  |
| Overweight | 2.55 (0.91-7.12) | 1.77 (0.48-6.50) |  |
| Obesity | **8.46 (2.81-25.42)** | 0.93 (0.17-5.20) | 0.062 |
| Self-perception of health status |  |  |  |
| Good, very good | Reference | Reference |  |
| Fair, poor, very poor | **6.28 (2.46-16.03)** | 2.52 (0.82-7.76) | 0.220 |
| **Lifestyles** |  |  |  |
| Adherence to the Mediterranean Diet (Reference: no) | 0.57 (0.21-1.56) | 0.30 (0.06-1.49) | 0.513 |
| Smoking |  |  |  |
| Never | Reference | Reference |  |
| Ever | 0.94 (0.38-2.29) | 8.02 (0.99-65.10) | 0.064 |
| Alcoholic beverages consumption (last 12 months) |  |  |  |
| Never or <1/month | Reference | Reference |  |
| ≥1/month to <1/day | 1.51 (0.63-3.63) | 0.27 (0.04-1.87) |  |
| ≥1/day | 0.36 (0.13-1.03) | **0.18 (0.03-1.00)** | 0.247 |
| Physical activity |  |  |  |
| Moderate/high | Reference | Reference |  |
| Low | 2.06 (0.98-4.32) | 1.09 (0.41-2.92) | 0.314 |
| CI, Confidence Interval; OR, Odds Ratio.  ^a^ Adjusted for age and years of schooling (continuous variables).  Note: Bold values represent p-values <0.050. | |  |  |

**Table S2** – Association between sociodemographics, health-related characteristics and lifestyles and having any problem in the self-care dimension, according to sex.

|  | **Any problem in the self-care dimension** | | | |
| --- | --- | --- | --- | --- |
|  | **Females** | **Males** | **p for**  **interaction** | |
|  | OR (95% CI)^a^ | OR (95% CI)^a^ |  |  |
| **Sociodemographic characteristics** |  |  |  | |
| Age (years) | 1.02 (0.92-1.11) | 1.00 (0.88-1.13) | 0.848 | |
| Education (years) |  |  |  | |
| 4 | Reference | Reference | ---- | |
| 5-9 | 0.79 (0.18-3.53) | ---- |  |  |
| ≥10 | 0.47 (0.09-2.52) | ---- |  |  |
| Marital status |  |  |  | |
| With partner | Reference | Reference | ---- | |
| Without partner | **4.75 (1.17-19.29)** | ---- |  |  |
| Occupational status |  |  |  | |
| Unemployed | Reference | Reference | ---- | |
| Employed | 1.73 (0.14-21.21) | ---- |  |  |
| Household income (€/month) |  |  |  | |
| ≤1000 | Reference | Reference | 0.945 | |
| 1001-1500 | 0.18 (0.02-1.58) | 0.28 (0.02-3.42) |  |  |
| 1501-2000 | 0.66 (0.11-3.87) | 0.44 (0.03-5.65) |  |  |
| >2000 | 0.31 (0.03-3.15) | 0.61 (0.04-8.47) |  |  |
| Social support |  |  |  | |
| Poor | Reference | Reference | 0.435 | |
| Moderate | 0.19 (0.03-1.28) | ---- |  |  |
| Strong | 1.24 (0.27-5.83) | 0.46 (0.06-3.36) |  |  |
| **Health-related characteristics** |  |  |  | |
| Previous diagnosis of health conditions (Reference: no) |  |  |  | |
| Hypertension | 2.03 (0.55-7.46) | 2.92 (0.31-27.79) | 0.784 | |
| Hypercholesterolemia | 1.83 (0.36-9.36) | 1.82 (0.18-18.78) | 0.997 | |
| Diabetes Type II | 1.31 (0.24-7.03) | 2.93 (0.43-19.88) | 0.535 | |
| Cardiovascular diseases | 1.01 (0.20-5.37) | 2.33 (0.30-17.88) | 0.539 | |
| Cancer | 0.80 (0.09-6.93) | **8.11 (1.19-55.16)** | 0.160 | |
| Respiratory diseases | 3.78 (0.95-15.01) | 0.56 (0.06-5.49) | 0.183 | |
| Musculoskeletal and connective tissue disorders | 1.92 (0.36-10.24) | ---- | ---- | |
| Renal and urinary disorders | **4.13 (1.09-15.61)** | **16.84 (1.70-166.77)** | 0.298 | |
| Gastrointestinal and hepatobiliary disorders | 1.69 (0.44-6.44) | 1.82 (0.23-14.12) | 0.955 | |
| Multimorbidity (Reference: no) | **----** | ---- | ---- | |
| Possible presence of cognitive impairment (Reference: no) | **6.84 (1.81-25.80)** | 0.64 (0.07-6.18) | 0.078 | |
| Body mass index |  |  |  | |
| Normal | Reference | Reference |  | |
| Overweight | 1.24 (0.11-14.40) | ---- |  | |
| Obesity | **8.93 (1.04-76.97)** | 2.14 (0.29-15.57) | 0.338 | |
| Self-perception of health status |  |  |  | |
| Good, very good | Reference | Reference |  | |
| Fair, poor, very poor | 4.42 (0.53-36.69) | 2.16 (0.23-230.58) | 0.648 | |
| **Lifestyles** |  |  |  | |
| Adherence to the Mediterranean Diet (Reference: no) | 0.44 (0.05-3.68) | ---- | ---- | |
| Smoking |  |  |  | |
| Never | Reference | Reference |  | |
| Ever | 1.06 (0.19-6.02) | **----** | ---- | |
| Alcoholic beverages consumption (last 12 months) |  |  |  | |
| Never or <1/month | Reference | Reference |  | |
| ≥1/month to <1/day | 0.31 (0.06-1.76) | 0.31 (0.06-1.76) |  | |
| ≥1/day | 0.71 (0.15-3.31) | 0.14 (0.02-1.11) | 0.216 | |
| Physical activity |  |  |  | |
| Moderate/high | Reference | Reference |  | |
| Low | 2.31 (0.63-8.48) | 4.59 (0.48-43.56) | 0.604 | |
| CI, Confidence Interval; OR, Odds Ratio.  ^a^ Adjusted for age and years of schooling (continuous variables).  Note: Bold values represent p-values <0.050. | | | |  |

**Table S3** – Association between sociodemographics, health-related characteristics and lifestyles and having any problem in the usual activities dimension, according to sex.

|  | **Any problem in the usual activities dimension** | | |  |
| --- | --- | --- | --- | --- |
|  | **Females** | **Males** | **p for**  **interaction** |  |
|  | OR (95% CI)^a^ | OR (95% CI)^a^ |  |  |
| **Sociodemographic characteristics** |  |  |  |  |
| Age (years) | 1.00 (0.95-1.06) | 1.04 (0.97-1.11) | 0.403 |  |
| Education (years) |  |  |  |  |
| 4 | Reference | Reference | 0.806 |  |
| 5-9 | 0.65 (0.24-1.75) | 0.40 (0.13-1.20) |  |  |
| ≥10 | 0.68 (0.26-1.76) | 0.60 (0.17-2.15) |  |  |
| Marital status |  |  |  |  |
| With partner | Reference | Reference | 0.484 |  |
| Without partner | 2.54 (1.13-5.71) | 1.05 (0.10-11.09) |  |  |
| Occupational status |  |  |  |  |
| Unemployed | Reference | Reference | 0.274 |  |
| Employed | 2.35 (0.61-9.08) | 0.60 (0.06-5.91) |  |  |
| Household income (€/month) |  |  |  |  |
| ≤1000 | Reference | Reference | 0.879 |  |
| 1001-1500 | 0.43 (0.15-1.26) | 0.46 (0.12-1.73) |  |  |
| 1501-2000 | 0.48 (0.14-1.66) | 0.24 (0.05-1.20) |  |  |
| >2000 | 0.43 (0.13-1.48) | 0.48 (0.10-2.16) |  |  |
| Social support |  |  |  |  |
| Poor | Reference | Reference | 0.966 |  |
| Moderate | 0.45 (0.15-1.33) | 0.38 (0.08-1.75) |  |  |
| Strong | 0.86 (0.28-2.65) | 0.66 (0.14-3.05) |  |  |
| **Health-related characteristics** |  |  |  |  |
| Previous diagnosis of health conditions (Reference: no) |  |  |  |  |
| Hypertension | 1.62 (0.73-3.59) | 1.56 (0.58-4.27) | 0.955 |  |
| Hypercholesterolemia | 1.55 (0.62-3.88) | 0.70 (0.26-1.92) | 0.253 |  |
| Diabetes Type II | **3.79 (1.26-11.36**) | 1.97 (0.60-6.42) | 0.426 |  |
| Cardiovascular diseases | 1.97 (0.74-5.20) | 1.40 (0.49-4.05) | 0.645 |  |
| Cancer | 0.61 (0.16-2.33) | 1.28 (0.38-4.32) | 0.422 |  |
| Respiratory diseases | **2.49 (1.03-5.97)** | 1.00 (0.32-3.13) | 0.214 |  |
| Musculoskeletal and connective tissue disorders | 1.13 (0.46-2.79) | 1.13 (0.31-4.16) | 0.996 |  |
| Renal and urinary disorders | **3.22 (1.32-7.85)** | 2.74 (0.89-8.47) | 0.843 |  |
| Gastrointestinal and hepatobiliary disorders | 1.50 (0.64-3.51) | 1.31 (0.44-3.86) | 0.842 |  |
| Multimorbidity (Reference: no) | **2.10 (0.42-10.40)** | 1.81 (0.35-9.43) | 0.900 |  |
| Possible presence of cognitive impairment (Reference: no) | 2.11 (0.83-5.39) | 1.11 (0.39-3.21) | 0.374 |  |
| Body mass index |  |  |  |  |
| Normal | Reference | Reference |  |  |
| Overweight | 1.11 (0.33-3.77) | 0.96 (0.25-3.64) |  |  |
| Obesity | **8.70 (2.72-27.73)** | **5.59 (1.17-26.66)** | 0.892 |  |
| Self-perception of health status |  |  |  |  |
| Good, very good | Reference | Reference |  |  |
| Fair, poor, very poor | **8.03 (2.26-28.48)** | 2.84 (0.93-8.69) | 0.227 |  |
| **Lifestyles** |  |  |  |  |
| Adherence to the Mediterranean Diet (Reference: no) | 0.54 (0.17-1.74) | **0.11 (0.01-0.93)** | 0.208 |  |
| Smoking |  |  |  |  |
| Never | Reference | Reference |  |  |
| Ever | 1.30 (0.50-3.40) | 1.42 (0.41-4.97) | 0.911 |  |
| Alcoholic beverages consumption (last 12 months) |  |  |  |  |
| Never or <1/month | Reference | Reference |  |  |
| ≥1/month to <1/day | 0.46 (0.17-1.22) | 0.18 (0.03-1.34) |  |  |
| ≥1/day | 0.40 (0.13-1.21) | 0.25 (0.05-1.30) | 0.724 |  |
| Physical activity |  |  |  |  |
| Moderate/high | Reference | Reference |  |  |
| Low | 2.58 (1.14-5.86) | 2.10 (0.80-5.57) | 0.748 |  |
| CI, Confidence Interval; OR, Odds Ratio.  ^a^ Adjusted for age and years of schooling (continuous variables).  Note: Bold values represent p-values <0.050. | | | | |

**Table S4** – Association between sociodemographics, health-related characteristics and lifestyles and having any problem in the pain/discomfort dimension, according to sex.

|  | **Any problem in the pain/discomfort dimension** | | |
| --- | --- | --- | --- |
|  | **Females** | **Males** | **p for**  **interaction** |
|  | OR (95% CI)^a^ | OR (95% CI)^a^ |  |
| **Sociodemographic characteristics** |  |  |  |
| Age (years) | 0.99 (0.94-1.04) | 1.02 (0.96-1.08) | 0.404 |
| Education (years) |  |  |  |
| 4 | Reference | Reference | 0.501 |
| 5-9 | 0.42 (0.16-1.12) | 0.69 (0.26-1.85) |  |
| ≥10 | 0.62 (0.23-1.65) | 0.40 (0.12-1.38) |  |
| Marital status |  |  |  |
| With partner | Reference | Reference | 0.186 |
| Without partner | 0.94 (0.43-2.10) | 5.12 (0.48-54.46) |  |
| Occupational status |  |  |  |
| Unemployed | Reference | Reference | 0.602 |
| Employed | 1.23 (0.33-4.56) | 2.03 (0.39-10.54) |  |
| Household income (€/month) |  |  |  |
| ≤1000 | Reference | Reference | 0.698 |
| 1001-1500 | 0.97 (0.30-3.08) | 0.33 (0.09-1.24 |  |
| 1501-2000 | 0.56 (0.16-1.93) | 0.29 (0.07-1.24) |  |
| >2000 | 0.61 (0.18-2.05) | 0.32 (0.08-1.41) |  |
| Social support |  |  |  |
| Poor | Reference | Reference | 0.155 |
| Moderate | 1.03 (0.32-3.33) | **0.10 (0.01-0.85)** |  |
| Strong | 0.82 (0.24-2.83) | **0.08 (0.01-0.69)** |  |
| **Health-related characteristics** |  |  |  |
| Previous diagnosis of health conditions (Reference: no) |  |  |  |
| Hypertension | 0.99 (0.45-2.17) | 1.33 (0.54-3.25) | 0.626 |
| Hypercholesterolemia | 1.61 (0.70-3.69) | 2.04 (0.51-5.15) | 0.710 |
| Diabetes Type II | 2.87 (0.61-13.54) | 1.08 (0.35-3.34) | 0.314 |
| Cardiovascular diseases | 2.34 (0.73-7.59) | 2.24 (0.84-5.99) | 0.950 |
| Cancer | 2.85 (0.61-13.39) | 1.59 (0.51-4.98) | 0.552 |
| Respiratory diseases | **3.45 (1.25-9.54)** | 1.53 (0.54-4.33) | 0.270 |
| Musculoskeletal and connective tissue disorders | 2.18 (0.93-5.14) | 0.74 (0.22-2.50) | 0.152 |
| Renal and urinary disorders | **5.52 (1.53-19.96)** | 2.14 (0.72-6.33) | 0.270 |
| Gastrointestinal and hepatobiliary disorders | **2.86 (1.10-7.45)** | 1.28 (0.47-3.47) | 0.254 |
| Multimorbidity (Reference: no) | **4.25 (1.34-13.45)** | 1.70 (0.46-6.32) | 0.297 |
| Possible presence of cognitive impairment (Reference: no) | 0.73 (0.28-1.92) | 0.58 (0.21-1.59) | 0.747 |
| Body mass index |  |  |  |
| Normal | Reference | Reference |  |
| Overweight | 1.65 (0.64-4.22) | **10.61 (2.15-52.50**) |  |
| Obesity | **3.04 (1.04-8.88)** | 5.15 (0.88-33.78) | 0.082 |
| Self-perception of health status |  |  |  |
| Good, very good | Reference | Reference |  |
| Fair, poor, very poor | **6.63 (2.77-15.86)** | 1.13 (0.46-2.81) | **0.006** |
| **Lifestyles** |  |  |  |
| Adherence to the Mediterranean Diet (Reference: no) | 0.69 (0.25-1.91) | 0.88 (0.29-2.65) | 0.742 |
| Smoking |  |  |  |
| Never | Reference | Reference |  |
| Ever | 0.95 (0.38-2.39) | 0.96 (0.33-2.82) | 0.981 |
| Alcoholic beverages consumption (last 12 months) |  |  |  |
| Never or <1/month | Reference | Reference |  |
| ≥1/month to <1/day | 1.07 (0.40-2.86) | 0.37 (0.06-2.33) |  |
| ≥1/day | 0.48 (0.17-1.34) | 0.50 (0.10-2.52) | 0.357 |
| Physical activity |  |  |  |
| Moderate/high | Reference | Reference |  |
| Low | 1.67 (0.74-3.75) | 1.86 (0.76-4.54) | 0.856 |
| CI, Confidence Interval; OR, Odds Ratio.  ^a^ Adjusted for age and years of schooling (continuous variables).  Note: Bold values represent p-values <0.050. | | | |

**Table S5** – Association between sociodemographics, health-related characteristics and lifestyles and having any problem in the anxiety/depression dimension, according to sex.

|  | **Any problem in the anxiety/depression dimension** | | |
| --- | --- | --- | --- |
|  | **Females** | **Males** | **p for**  **interaction** |
|  | OR (95% CI)^a^ | OR (95% CI)^a^ |  |
| **Sociodemographic characteristics** |  |  |  |
| Age (years) | 1.01 (0.96-1.06) | 0.99 (0.94-1.05) | 0.602 |
| Education (years) |  |  |  |
| 4 | Reference | Reference | 0.747 |
| 5-9 | 0.47 (0.18-1.20) | 0.79 (0.29-2.12) |  |
| ≥10 | 0.54 (0.21-1.36) | 0.72 (0.22-2.39) |  |
| Marital status |  |  |  |
| With partner | Reference | Reference | 0.570 |
| Without partner | 0.88 (0.41-1.89) | 0.43 (0.04-4.45) |  |
| Occupational status |  |  |  |
| Unemployed | Reference | Reference | 0.458 |
| Employed | 0.83 (0.24-2.95) | 0.39 (0.06-2.39) |  |
| Household income (€/month) |  |  |  |
| ≤1000 | Reference | Reference | 0.722 |
| 1001-1500 | 1.64 (0.57-4.76) | 0.69 (0.20-2.44) |  |
| 1501-2000 | 1.24 (0.38-4.07) | 0.55 (0.14-2.19) |  |
| >2000 | 1.80 (0.55-5.90) | 0.85 (0.20-3.53) |  |
| Social support |  |  |  |
| Poor | Reference | Reference | 0.490 |
| Moderate | **0.09 (0.01-0.74)** | 0.43 (0.09-2.10) |  |
| Strong | **0.10 (0.01-0.83)** | 0.46 (0.10-2.22) |  |
| **Health-related characteristics** |  |  |  |
| Previous diagnosis of health conditions (Reference: no) |  |  |  |
| Hypertension | 1.52 (0.71-3.26) | 0.79 (0.32-1.91) | 0.268 |
| Hypercholesterolemia | 0.80 (0.35-1.81) | 0.57 (0.23-1.41) | 0.594 |
| Diabetes Type II | 0.66 (0.22-2.02) | 1.99 (0.63-6.29) | 0.177 |
| Cardiovascular diseases | 1.02 (0.39-2.67) | 2.86 (1.06-7.72) | 0.145 |
| Cancer | 2.17 (0.57-8.18) | 0.53 (0.16-1.75) | 0.124 |
| Respiratory diseases | 0.88 (0.38-2.02) | 1.07 (0.38-3.01) | 0.764 |
| Musculoskeletal and connective tissue disorders | 1.42 (0.61-3.27) | 1.339 (0.43-4.51) | 0.976 |
| Renal and urinary disorders | **3.35 (1.15-9.77)** | 2.14 (0.72-6.37) | 0.566 |
| Gastrointestinal and hepatobiliary disorders | 1.70 (0.73-3.97) | 1.12 (0.42-2.99) | 0.529 |
| Multimorbidity (Reference: no) | 0.89 (0.28-2.81) | 1.23 (0.35-4.32) | 0.706 |
| Possible presence of cognitive impairment (Reference: no) | 1.63 (0.59-4.53) | 1.90 (0.71-5.07) | 0.832 |
| Body mass index |  |  |  |
| Normal | Reference | Reference |  |
| Overweight | 1.51 (0.58-3.95) | 1.71 (0.53-5.50) |  |
| Obesity | 0.73 (0.28-1.89**)** | 2.14 (0.50-9.15) | 0.380 |
| Self-perception of health status |  |  |  |
| Good, very good | Reference | Reference |  |
| Fair, poor, very poor | 0.91 (0.40-2.05) | 1.88 (0.75-4.73) | 0.246 |
| **Lifestyles** |  |  |  |
| Adherence to the Mediterranean Diet (Reference: no) | 1.21 (0.43-3.43) | 0.43 (0.13-1.37) | 0.190 |
| Smoking |  |  |  |
| Never | Reference | Reference |  |
| Ever | 1.68 (0.66-4.27) | 1.92 (0.63-5.85) | 0.860 |
| Alcoholic beverages consumption (last 12 months) |  |  |  |
| Never or <1/month | Reference | Reference |  |
| ≥1/month to <1/day | 0.78 (0.31-1.97) | 0.36 (0.06-2.27) |  |
| ≥1/day | 0.88 (0.31-2.48) | 0.70 (0.14-3.50) | 0.705 |
| Physical activity |  |  |  |
| Moderate/high | Reference | Reference |  |
| Low | 0.62 (0.29-1.32) | **2.60 (1.05-6.39)** | **0.017** |
| CI, Confidence Interval; OR, Odds Ratio.  ^a^ Adjusted for age and years of schooling (continuous variables).  Note: Bold values represent p-values <0.050. | | | |
